# New insights into the prevention of ureteral stents encrustation

**DOI:** 10.1515/med-2023-0854

**Published:** 2023-12-06

**Authors:** Hao Guo, Jun-Bin Yuan

**Affiliations:** Department of Urology, Xiangya Hospital, Central South University, Changsha, 410078, Hunan Province, PR China

**Keywords:** ureteral stents, encrustation, conditioning film, biological materials, prevention

## Abstract

Ureteral stents are commonly used medical devices for the treatment of urinary system diseases. However, while providing benefits to patients, they often give rise to various issues, with stent encrustation being a major concern for clinical physicians. This phenomenon involves the formation of attached stones on the stent’s surface, leading to potential complications such as increased fragility and laxity of the ureter, difficulties in stent removal, and a higher risk of stent fracture. Therefore, this review starts from the pathological mechanisms of stone formation and discusses in detail the two major mechanisms of stent encrustation: the conditioning film and the biofilm pathway. It also examines multiple risk factors associated with ureteral stents and patients. Furthermore, the review updates the research progress on the structure, materials, and bio-coatings of ureteral stents in the prevention and treatment of stent encrustation. It presents new insights into the prevention and treatment of stent encrustation. This includes individualized and comprehensive clinical guidance, the use of novel materials, and early intervention based on physiological and pathological considerations. Ultimately, the study offers an encompassing overview of the advancements in research within this field and provides the latest insights into strategies for preventing and treating stent encrustation.

## 1 A double-edged sword: the ureteral stent

The urinary system, as one of the eight major systems in the human body, comprises vital organs such as the kidneys, ureters, and bladder. Due to its essential role in metabolism and excretion, diseases of the urinary system often have systemic implications, emphasizing the significance of maintaining normal urinary system function for overall health [1]. Among these, the ureters are an important component of the urinary system and are susceptible to various factors such as high-salt diet, inadequate fluid intake, and high-protein diet, leading to the occurrence of diseases in this position [2]. Currently, common clinical conditions affecting the ureters include urinary tract stones and ureteral infections [3]. Meanwhile, abnormalities in ureteral function often give rise to associated complications that impact both the kidneys and the bladder [4].

The ureteral stent, commonly known as a Double-J or Pig-tail stent, is widely used in urological conditions, particularly for patients with ureteral or kidney stones, as well as those with strictures, to enhance their quality of life [5]. The primary function of a ureteral stent is to provide support, dilation, and drainage of the ureter. This includes expanding the ureter to relieve obstructions caused by inflammatory swelling, repairing strictures in the ureter, and resolving ureteral blockages due to stones [6]. Urologists consider it an indispensable medical device after performing various urological surgical procedures to stabilize the ureter’s physiological function and prevent temporary strictures resulting from inflammation or blood clots. However, the use of ureteral stents is a double-edged sword that can lead to complications, significantly impacting the effectiveness of treatment and the patients’ quality of life [7]. Some of the complications associated with ureteral stents include stent encrustation, rupture, displacement, pain [8], discomfort [9], infection, and hematuria [7,10]. These complications highlight the need for careful consideration and management when using ureteral stents to ensure that patients receive optimal treatment outcomes while minimizing potential adverse effects.

## 2 A frustrating dilemma: ureteral stents encrustation

In the realm of extensive research, one particularly daunting challenge for clinical physicians arises from ureteral stent encrustation, a problem that affects a substantial proportion of patients, with reported occurrence rates reaching up to 21.6% [11]. Encrustation, which is the deposition of mineral crystals, with calcium oxalate being the most common component (43.8%), primarily in hydrate form (27.1%), followed by protein (27.4%), calcium phosphate (16.4%), uric acid (5.2%), and struvite (2.9%) occurs on the surface and lumen of a ureteral stent [12]. The occurrence of encrustation on a ureteral stent results in the attachment of stones, making the stent more brittle, reducing its tensile strength, and increasing the difficulty of removal, thereby elevating the risk of stent fracture [13]. Therefore, the pain that ureteral stent encrustation can inflict on patients in the delicate and sensitive environment of the ureter is unimaginable. Faced with such a predicament, patients often have no choice but to undergo ureteroscopy or even open surgery for management, causing significant challenges to clinicians and further intensifies the patients’ pain. So far, despite the existence of comprehensive clinical strategies, the best solution is still to prevent encrustation of ureteral stents, thereby avoiding secondary surgeries and maximizing patient benefits [14].

As for the classification of stent systems, Manzo et al. proposed a visual classification system of stent structures in which visual grading for ureteral encrusted stent (V-GUES) classification system are graded from A to D, increasing with the severity of encrustation [15]. And it was thought that V-GUES classification can help patients get the best treatment options and outcomes. However, the more mainstream recognition is the FECal (forgotten, encrusted, calcified) ureteral stent grading system developed by Acosta-Mirand to assess the severity of stent encrustation at present. In this system, grade I corresponds to encrustation limited to the distal caudal part of the stent, specifically the bladder. Grade II indicates encrustation in the proximal caudal part, which is the renal pelvis. Grade III signifies encrustation in both the proximal caudal and intermediate segments of the stent, involving the renal pelvis and ureter. Grade IV represents encrustation in both the proximal and distal caudal parts, affecting the renal pelvis and bladder. Finally, Grade V describes a stent completely covered with a crust. A similar grading system is also utilized, known as the KUB system [16].

In cases where a patient has a long-term indwelling ureteral stent or is experiencing clinical symptoms like hematuria, pain, and discomfort, it is recommended to conduct a kidney, ureter, and bladder X-ray before stent removal [17]. This X-ray helps determine the presence and extent of stent encrustation. If crusts are identified, the severity of encrustation is assessed, and the appropriate procedure is selected [18,19]. Generally, for patients with stent encrustation limited to the bladder (FECal I), the stent can be grasped using rigid forceps under cystoscopic guidance to fragment any attached stones on the distal coil of the stent. The stent is then clamped and gently withdrawn with fluoroscopic guidance. In cases of stent encrustation in the renal pelvis (FECal II), a guidewire can be inserted into the center of the stent to unfold the proximal curl, facilitating its removal. However, if the proximal curl cannot be straightened or the guidewire cannot pass through the crusted stent lumen, interventional techniques such as advancing a catheter sheath along the stent’s surface may be necessary. For encrustation in the ureteral portion of the stent (FECal III), retrograde ureteroscopy and holmium laser lithotripsy can be employed. It is advisable to leave a safety guidewire next to the crusted stent during the procedure to maintain ureteral access. If the retrograde ureteroscope cannot access the stent along the guidewire, the area with the highest density of attached stones should undergo extracorporeal shock wave lithotripsy before stent removal via ureteroscopy. In cases of severe proximal stent scaling or calcification (FECal IV, V), percutaneous nephrolithotomy combined with paracentesis may be preferred. This approach provides direct access to the renal pelvis, facilitating the removal of the proximally encrusted stent. Care should be taken to avoid forceful traction during stent removal to prevent ureteral injury, laceration, stent fracture, or residual fragments.

## 3 Retrospective reflection: how does ureteral stent encrustation start?

When addressing the issue of ureteral stent encrustation, the primary objective for clinical practitioners is to either lower the occurrence rate of encrustation or minimize its detrimental impact on patients, ultimately enhancing treatment outcomes. In essence, clinical researchers are dedicated to preventing the occurrence of encrustation. Consequently, gaining a comprehensive understanding of the pathological mechanisms underlying encrustation becomes a paramount priority.

### 3.1 Protein deposition on the conditioning film

The protein deposition theory is an earlier hypothesis, in which researchers believe that proteins on the conditioning film directly facilitate the attachment of substances in the urine to the stent through various mechanisms. This, in turn, creates conditions for the deposition of various compounds and proteins, leading to the aggregation and solidification of compounds such as proteins and calcium elements in the urine and tissues, ultimately forming encrustation [20,21]. While the exact mechanism is still being investigated, it is widely acknowledged that the conditioning film serves as the initial stage in the formation of stent encrustation.

This film is created through the adsorption of proteins and polysaccharide ions from urine onto the stent scaffold [22], typically occurring immediately after the stent placement [23]. In addition, some studies suggest that keratin from uroepithelial cells [24], as well as histone H2b and H3a [25] on the surface, seem to play a crucial role in the early development of the conditioning film. Subsequently, according to the Vroman effect [26], the small proteins attached to the stent at an earlier stage begin to adsorb larger proteins that are less mobile in the urine or tissue that can typically contain collagen, fibrinogen, albumin, etc. [27]. Surprisingly, studies on the conditioning film have found that its composition may include approximately 100 proteins, with the most common ones being cytokeratin isoforms, serum albumin, hemoglobin subunit beta, and uromodulin (Tamm-Horsfall protein) [24]. This finding greatly explains the frequent occurrence of encrustation, as the presence of a large number of these initial proteins provides a greater likelihood for the formation of encrustation.

Based on this, understanding the formation and mechanisms of the conditioning film has become an important breakthrough in research on the prevention and control of stent encrustation. Given the vital role of proteins in the conditioning film for stent encrustation, researchers initially focus on qualitative and quantitative analysis, as well as the chemical characteristics and pathological relevance of proteins in the conditioning film, including their molecular size, charge, and chemical interactions, as these factors play crucial roles in the initiation of stent encrustation [24,28]. Among them, proteins such as alpha-1 antitrypsin, Ig kappa, IgH G1, and histones H2b and H3a showed a strong association with stent encrustation, while uromodulin and histone H2a had minimal effects in this context [25]. Meanwhile, highly positively charged proteins, such as histones H2b, H3, and H4, are believed to facilitate the aggregation of negatively charged crystals. Previous studies have proposed electrostatic effects, suggesting that plasma proteins like serum albumin, globulin, and fibrinogen present in the conditioning film also play a significant role in stent encrustation. These proteins interact with hydroxyapatite, which is negatively charged, through electrostatic interactions [29]. Additionally, calcium-binding proteins, including urinary regulator and S100A9 protein, have been found on the conditioning film and can adsorb calcium ions from urine, becoming the primary focus of wall stones in certain research studies [24].

### 3.2 Microorganisms on the biofilm

On the other hand, due to the semi-permeable nature of the ureter and its rich microbial environment [30], researchers have started to pay attention to the role of microorganisms in the inflammation and pain caused by ureteral stent encrustation [28,31]. Moreover, some studies have found that microorganisms on the biofilm mediate the precipitation of crystals, which may be another significant factor contributing to ureteral stent encrustation [32]. Given the unique adhesive properties of microorganisms and their involvement in numerous studies on stone formation [33], there have been extensive research reports on the role of biofilms in the formation of stent encrustation [25,34]. Biofilms are microbial communities composed of microorganisms and their secreted extracellular polymer matrix [35]. They serve various functions for microorganisms, including the ability to survive in adverse conditions [36]. For instance, bacteria within a biofilm exhibit resistance to antimicrobial agents, up to a thousand times more than their planktonic counterparts [37]. The development of biofilms is a continuous and complex process, with different mechanisms occurring on various surfaces [35]. In the context of ureteral stents, biofilm formation begins with the initiation of a conditioning film [38]. This film modifies the surface of the stent and provides attachment sites for bacterial adhesins [39]. Due to advancements in stent technology, many bacteria are unable to adhere directly to the implant, making the conditioning film crucial for biofilm formation [38,40].

And the key to biofilm formation is bacteria. Unfortunately, non-uremic patients often experience bacterial colonization after the insertion of simple indwelling ureteral stents [41]. Epidemiological studies have shown a direct relationship between the duration of stent retention and bacterial colonization. In fact, bacterial colonization occurs in approximately 87.5% of stents within 120 days of insertion [42]. The reason behind this phenomenon may be attributed to the elimination of the immune response from the bladder and ureteral mucosa, which occurs due to the presence of the stent. Consequently, bacteria can enter the urinary tract, leading to infections and eventually colonizing the stent through the “low resistance pathway” it provides [43]. It is important to note that while most patients with indwelling ureteral stents experience bacterial colonization or infection, it does not necessarily mean they will have bacteriuria (bacteria in the urine) [44]. Research conducted by Reid found bacterial colonization in 90% of stents, but only 27% of patients had bacteriuria [28]. When bacteria adhere to the conditioning film on the stent’s surface, they can multiply and form a biofilm. This biofilm enables microorganisms to float freely within it and spread across the stent’s surface [39].

Regarding the formation of stent encrustations, it is believed that urease-producing bacteria present in biofilms play a significant role. These bacteria include species such as Proteus, Klebsiella, Pseudomonas, and Staphylococcus [45]. Urease enzymes produced by these bacteria hydrolyze urea in urine, resulting in the production of ammonia and carbon dioxide [46]. This process alkalizes the urine, allowing ammonium ions to combine with phosphate and magnesium ions in the urine, leading to the formation of struvite calculi [47]. Additionally, bicarbonate in urine can combine with cations to form carbapatite [48,49]. However, it should be noted that the study mentioned in the previous statement found that only a small proportion (2.9%) of the attached stones were predominantly composed of struvite stones [12]. This low proportion may be due to the fact that struvite stones are often bound to calcium phosphate (primarily carbapatite) and calcium oxalate [50]. A retrospective study on the composition of infected stones revealed that only around 13% of stones were purely composed of struvite. The majority (about 87%) of struvite stones were found to be mixed with other mineral components, with calcium phosphate, calcium oxalate, calcium carbonate, and uric acid being the most abundant in that order [51].This suggests that while the early stages of stent encrustation may be dominated by the formation of struvite stones, in patients with a complex urine profile, the composition of the encrustations may subsequently shift toward being primarily composed of calcium oxalate-based stones.

Additionally, in cases where the bacteria are urease-negative, some researchers argue that the biofilm’s extracellular polysaccharides can retain crystals in the urine, leading to the formation of a single crust-like lesion [52]. Furthermore, non-urease-producing bacteria like *Escherichia coli* have been found to contribute to the development of calcium oxalate stones [50]. Animal studies have demonstrated that in polymicrobial urinary tract infections, regardless of urease production, the presence of other bacteria can enhance the urease activity of *Proteus mirabilis* and exacerbate the severity of the disease [53]. Overall, proponents of this second perspective believe that stent encrustation is an ongoing process. It begins with the formation of a conditioning film, which then progresses into a biofilm. Eventually, under the influence of bacteria, the biofilm can transform into a crystalline film. Crusting plays a role in promoting both biofilm formation and bacterial colonization [13], which are mutually dependent and reinforcing processes [52].

## 4 Risk factors for ureteral stent encrustation

Either protein deposition or microbial acceleration serve as a reminder that encrustation is a complex process. It typically necessitates the gradual accumulation of substances over a specific duration within a particular and conducive internal environment. This suggests that various factors can impact the progression of ureteral stent encrustation. First and foremost, it is crucial to acknowledge that time is a significant and formidable risk factor to consider. There is a direct correlation between the duration of stent retention and the risk of encrustation occurrence, making time management a challenging aspect to address. It is now widely accepted that prolonged retention is a significant risk factor for ureteral stent encrustation [13,54,55]. In 1991, El-Faqih et al. made the initial observation that polyurethane (PU) ureteral stents retained for less than 6 weeks had a 9.2% chance of developing encrustation. This probability increased to 47.5% for stents retained between 6 and 12 weeks, and further rose to 76.3% for stents retained longer than 12 weeks [56]. Recent studies have confirmed similar findings, reporting an incidence of encrustation of 26.8% for stents retained for less than 6 weeks, 56.9% for stents retained between 6 and 12 weeks, and 75.9% for stents retained for more than 12 weeks [55]. Although the precise timing of encrustation may vary, it is indisputable that the risk and severity of encrustation amplify with prolonged retention periods.

On the other hand, with the era of personalized medicine, clinicians are increasingly aware that individual differences manifest unique physiological and pathological characteristics in clinical practice [57]. For instance, different disease backgrounds in patients often have an impact on the process of ureteral stent encrustation [58]. Studies have shown that in patients with ureteral stents left in place after lithotripsy (a procedure to break up kidney stones), there is a high correlation between the composition of the original stones and the stent stones that develop later [59,60]. In fact, a significant proportion of stent stones (approximately 78%) have been found to have the same composition as the original stones [61]. In patients with urolithiasis, if the underlying metabolic abnormalities that contribute to stone formation, such as hyperoxaluria or hypercalciuria, are not addressed, the concentration of solutes in the urine can exceed their solubility. The placement of a ureteral stent can provide a new nucleus for stone growth and further promote the development of stent encrustation, where stone material accumulates on the stent surface [59]. In summary, urolithiasis patients who have not had the underlying metabolic abnormalities addressed may be at increased risk of stent encrustation and the formation of stent stones. The composition of these stones often reflects the composition of the original stones, with calcium oxalate stones being the most common type. Interestingly, it is not directly related to the level of calcium ions in the urine [34]. Instead, it is influenced by the concentration of a compound called C_2_H_4_, which is also known as ethylene and plays a role in the formation of crusts on urinary stones [62]. Similarly, research has shown that urinary tract infections can promote the development of calcium oxalate stones in individuals who are already sensitive to common uropathogenic bacteria such as *E. coli*, *Klebsiella*, and *Staphylococcus aureus* [50]. Urinary tract infections can also contribute to the formation of noninfectious stones [63]. Additionally, animal experiments have demonstrated that other urological pathogens, during polymicrobial urinary tract infections, can enhance the activity of urease in *P. mirabilis* and further promote the development of struvite stones [53]. Certain medical conditions, including diabetes, chronic renal failure, and gynecological diseases, are often associated with recurrent urinary tract infections. These conditions can increase the bacterial load in the urine, subsequently increasing the risk of stent crusting [64].

Individual physiological differences will naturally influence the formation of encrustation on ureteral stents, with age, gender, race, and other factors demonstrating certain variations [65,66]. For instance, older people are at a higher risk of developing encrustation with indwelling stents, which investigators speculate may be associated with bladder dysfunction [67], and potentially dyslipidemia as well [68]. Similarly, pregnant women are also at a higher risk, possibly due to pregnancy-related absorptive hypercalciuria and hyperuricemia [69]. Therefore, clinical physicians often need to timely remind patients about the appropriate timing for ureteral stent replacement or provide other specific precautions based on individual circumstances, rather than providing standardized advice to all patients [70].

## 5 Strategies of researchers: potential of new materials

Confronting the formidable challenge presented by ureteral stent encrustation, both clinical physicians and researchers are actively committed to finding effective solutions. Notably, substantial advancements have been achieved in the realm of design and technology concerning ureteral stents [5,71]. Considering that the key factors contributing to encrustation are protein deposition and bacterial colonization, research efforts primarily focus on inhibiting these processes [72,73]. This encompasses the development of new materials for stents [74], the emergence of stent coatings [75], and a particular focus on redesigning the stent architecture [76].

Currently, polyethylene, PU, silicone, and their derivatives are the most commonly used multi-polymer materials in clinical practice. PUs are a class of polymers that contain urethane characteristic units in their main chain. They exhibit a moderate level of hardness and softness, along with high tensile strength and retention, making them suitable for medical applications. Moreover, PUs demonstrate good resistance to the formation of crusts. There are two types of PUs: polyether and polyester. Among them, polyether is more susceptible to biodegradation, while polyester offers better stability in the body. Hence, PUs, particularly the polyester type, are more widely employed. Additionally, polyether-based materials have properties that discourage bacterial growth [77]. Meanwhile, silicon materials are extensively employed in clinical settings due to their remarkable biocompatibility, which renders them highly resistant to stone formation and biofilm development [78]. However, their notable drawbacks include an exceptionally high surface friction coefficient and a soft material composition, making them challenging to insert and significantly diminishing their practical utility [79]. Fortunately, recent research has discovered a potential solution to this issue by introducing a hydrophilic coating onto the surface of silicon materials. This coating not only mitigates the problem of insertion but also imparts enhanced flexibility compared to PU, thereby significantly reducing patient discomfort [80]. These advancements in silicon material technology have opened up new possibilities for their application in various clinical procedures. By addressing the challenges associated with their insertion, the hydrophilic coating has improved the usability and effectiveness of silicon materials, expanding their potential in medical interventions and patient care. Furthermore, researchers have found that materials with slight hydrophilicity (contact angle of approximately 85°) and a strong negative zeta potential (approximately −60 mV) are most suitable for use as ureteral stent materials, as they exhibit minimal deposition of crystalline substances on the biofilm [81]. Therefore, these factors can assist researchers in rapidly screening anti-crust materials.

In addition to multi-polymer materials, metallic materials are commonly utilized for stents, including stainless steel [74], zinc alloy materials [82,83], copper-containing materials [74], superalloy titanium, or nickel/titanium alloys. These stents are typically designed to be self-expanding, generating a greater lateral radial force compared to the compression exerted by the surrounding tissue after insertion. As a result, the stents become covered by the uroepithelium against the wall, effectively reducing the risk of stone formation on the stent surface. They are particularly well-suited for treating complex ureteral strictures where conventional stent placement is challenging, as well as for patients with compression due to adjacent tumors. However, metallic stents come with certain drawbacks. They are not easily removable once positioned and carry a higher probability of stent displacement, inward growth of fibroblastic or tumor tissue, epithelial hyperplasia, urethral or distal ureteral strictures, severe fibrosis, and secondary obstruction [84]. Despite their advantages, these drawbacks underscore the need for careful consideration and assessment when opting for metallic stents in clinical scenarios.

The purpose of applying additional coatings to ureteral stents is to reduce the formation of biofilms and attached stones, as well as to improve the ease of insertion by reducing the coefficient of friction of the biomaterial. Calcium oxalate deposition has been considered to be an important factor in encrustation. Oxalate-degrading enzymes from *Oxalobacter formigenes* is a novel device coating to reduce urinary tract biomaterial-related encrustation [85]. HydroPlus^®^, composed of hydrophilic polymorphs, draws water into its structure, leading to a decrease in the biomaterial’s friction coefficient and an enhancement in biocompatibility by reducing frictional irritation between the stent and the ureter [86]. Additionally, the hydrogel coating naturally peels off shortly after stent insertion, preventing the formation of a conditioning film during this period. This dynamic surface property that constantly changes may be beneficial in slowing down the development of crust [68]. In Yao et al.’s study, a metal-catechol-assisted mussel chemistry was employed for surface functionalization of commercially available catheters with antimicrobial peptides, for the purpose of long-term anti-infection and encrustation prevention. To improve the stent stability, they used Cu^2+^-coordinated dopamine self-polymerization coating and determined that it had better stability and antibacterial effect [87]. In another similar study, Cottone found that 2-hydroxyethyl methacrylate-coated pellethane thermoplastic PU (TPU) and tetraethylene glycol dimethyl ether-coated pellethane TPU shows less encrustation tendency and has the potential to be a favorable substitute for traditional urethral stent materials [88]. It was found that cationic polyethyleneimine (PEI) brushes grafted on PU stents can reduce tissue inflammation and biofilm formation and crusting [89]. Other coating options include heparin coatings (Radiance^®^), diamond-like coatings (VisioSafe DIAMOND^®^), cationic PEI [90], silver sulfadiazine coated stents [91], poly(*N*,*N*-dimethylacrylamide) coatings [91], and others. These coatings, to varying extents, achieve similar objectives as mentioned above. The mechanism behind anti-crusting properties may involve the negatively charged surface of the coating, which inhibits the aggregation of negatively charged crystals, as seen in the case of heparin coatings [92]. In the case of diamond-like coatings, researchers speculate that this chemically inert substance seems to prevent the formation of conditioning films or inhibit bacterial growth [93].

Although numerous efforts have been made to enhance stent structures, they have largely proven ineffective in reducing the incidence of crusting. Most modifications have been primarily focused on alleviating patient discomfort. For example, the single pigtail suture stent, which lacks a “J” shape at its lower end and is predominantly composed of a softer material, has shown potential in reducing bladder irritation symptoms in patients [94,95]. Nevertheless, specific findings have brought additional insights. One study observed a higher crusting rate with Percuflex^®^ stents smaller than 6F and a lower encrustation rate with stents larger than 7F [56]. This disparity could be attributed to the enhanced urine flow and reduced stasis associated with larger stents. However, another study found no correlation between stent length or patency and the risk of encrustation [55]. Importantly, the spiral-ridged ureteric stents, aimed to maintain the integrity of the ureteral lumen and enable free urine flow in the ureter lumen enclosing the spiral, are also applied innovatively. Some research studies proved that spiral stents significantly reduced patient analgesic requirements in the clinical trail [96], and total flow past the spiral stent was significantly greater than flow with the smooth-walled stent under all conditions tested [97]. Moreover, porous chitosan ureteral stents were proved to have high stent resilience, effective viscosities, and the effectiveness of the radially aligned porosity for drainage [98]. Currently, ongoing investigations are exploring innovative stent structures to ascertain their potential benefits for patients [99].

## 6 Encrustation: comprehensive control perspective

**Figure 1:**
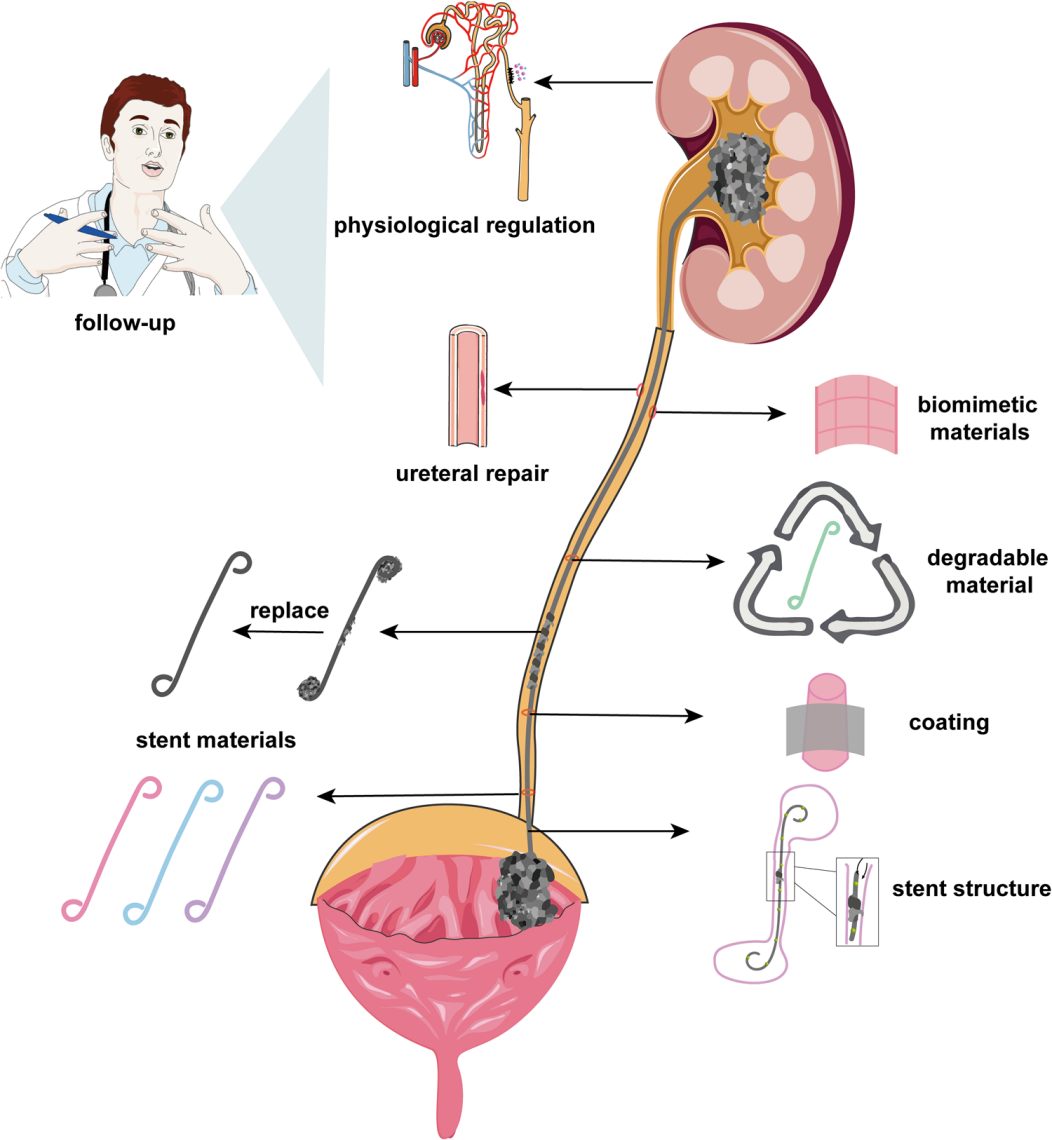
Comprehensive approach to ureteral stents encrustation prevention. The attention of clinicians included the regulation of patients’ physiology and pathology, the guidance of ureteral recovery, and the strategy of stent replacement. The design of stents structure, stents materials, surface coating, and some new materials including biodegradable materials and bionic materials will be the potential prevention strategy of stent stone.

First and foremost, we need to acknowledge that, based on the current clinical situation, the primary principle for the use of ureteral stents is to address clinical issues, followed by reducing patient discomfort to achieve a better quality of life. The exploration of ureteral stent encrustation should be approached from a more comprehensive perspective. So we provided comprehensive approach to ureteral stents encrustation prevention (Figure 1). It is believed that the prevention and treatment of ureteral stent encrustation should be a shared desire among all doctors, researchers, and patients. The current strategies for addressing encrustation still primarily focus on the stent itself, including improvements in stent materials, modifications to the structure, and the design of surface coatings, which have achieved certain results at different levels. However, these approaches have not fully met the objectives of current prevention and treatment efforts.

A new perspective shared by basic researchers and healthcare professionals is that this issue requires collaborative efforts. First, in terms of the treatment and adjunctive strategies for urological diseases, particularly the use of ureteral stents, it is important for clinical physicians to focus more on the maintenance and treatment strategies for the ureter itself, rather than simply relying on stents as a temporary solution. The long-term placement of ureteral stents within the body will eventually become obsolete. Second, in the current context of ureteral stent usage, material development remains a key area of focus. The desired characteristics for stent materials, such as biocompatibility, flexibility, compressive strength, antimicrobial properties, and low adhesion, are crucial for meeting patient needs [100,101]. The comfort of the stent’s structure, smoothness for unimpeded urine flow, and potential risks of frictional effects on the urothelium should also be taken into account. Moreover, the use of coating technologies should be approached with caution, considering the advantages of appropriate coating materials for encrustation prevention in terms of safety, tissue compatibility, non-deposition, and non-dispersion [102,103]. Another innovative approach involves the development of biodegradable materials [104], which, aided by advancements in bioengineering, offer new possibilities for ureteral stent development. The concept revolves around designing stents that will gradually dissolve in urine over time, eliminating the need for stent removal or secondary surgeries and mitigating associated complications. This idea is indeed enticing. In extreme cases where the patient’s ureter is extremely fragile, tissue reconstruction using biomimetic materials may be a more suitable alternative to ureteral stents. Other valuable suggestions include clinicians paying more attention to patients’ individual concerns and considering the pathophysiological characteristics that contribute to encrustation risk. Guiding patients toward a healthy diet, physiological activity regulation, and improved kidney function are important aspects that cumulatively benefit patients as a whole [105]. For example, it has been reported that taking potassium citrate after ureteral stent implantation can significantly reduce the formation of calcium oxalate and uric acid scabs on the stent, which has a positive indication for preventing stent scabs [106,107]. Furthermore, for patients requiring ureteral stent replacement, physicians should consider encrustation and other factors when selecting an appropriate stent material that facilitates easy replacement, and they should advise patients to undergo regular examinations for timely replacement. Overall, clinicians need to make individualized decisions in selecting a more appropriate ureteral stent for their patients, taking into account their specific diseases or conditions.

## 7 Conclusion

Although the primary purpose of ureteral stent placement is to alleviate symptoms resulting from urologic surgery, the occurrence of stent encrustation can significantly impact a patient’s quality of life and recovery. Stents that become encrusted are often referred to as “forgotten stents,” highlighting the importance of prevention over treatment and the need for clinical vigilance in managing this condition.

Currently, extensive research is being conducted on preventing ureteral encrustation, with a particular focus on antimicrobial stent materials or coatings [108]. Furthermore, clinicians have shown interest in exploring novel biodegradable or resorbable materials. However, effectively addressing the issue of ureteral stent encrustation requires collaboration among clinical, basic, and engineering researchers. One of the primary challenges is the limited understanding of the underlying causes of encrustation. Therefore, it is crucial for novel stent concepts to prioritize the benefits and well-being of patients. Clinicians should also consider the individualized status of each patient, taking into account their unique concerns and circumstances.

In conclusion, if more high-quality research articles become available in the future to further investigate the exact mechanism of ureteral stent encrustation, it will make a significant contribution to the development of appropriate prevention and treatment measures, as well as the design of effective anti-encrustation stents. In conclusion, if more high-quality research articles become available in the future to further investigate the exact mechanism of ureteral stent encrustation, it will make a significant contribution to the development of appropriate prevention and treatment measures, as well as the design of effective anti-encrustation stents. However, it is important to recognize the complexity of urine composition and the interactions of ureteral stents with various organic substances in the body. Therefore, conducting comprehensive studies in this field is expected to face challenges.
